# Analysis of “Hybrid Closed Loop Using a Do-It-Yourself Artificial Pancreas System in Adults With Type 1 Diabetes”

**DOI:** 10.1177/19322968231208216

**Published:** 2023-10-18

**Authors:** Tom M. Wilkinson, Martin de Bock

**Affiliations:** 1 Department of Paediatrics, University of Otago, Christchurch, Christchurch, New Zealand

**Keywords:** open-source, automated insulin delivery

## Abstract

In an article in *Journal of Diabetes Science and Technology*, Nanayakkara and colleagues assessed the glycemic efficacy and safety of AndroidAPS, an open-source automated delivery (AID) system, in a crossover randomized controlled trial. Although the trial included only 20 participants during a relatively short 4-week intervention period, glycemic outcomes attained were similar to commercial AID systems and there were no safety concerns. Validation of open-source AID systems in studies such as this should help address clinician hesitancy regarding these systems, and affirms the role of patient-centered innovation and self-management in diabetes care.

Type 1 diabetes (T1D) carries a unique burden of self-management. Regardless of treatment modality, people living with T1D interact with diabetes and make active treatment decisions multiple times per day, every day. Diabetes management is benefited when people with T1D are personally empowered to take management decisions themselves, rather than being reliant on intermittent interactions with healthcare professionals.

Ongoing improvements in continuous glucose monitoring (CGM) systems have enabled the development of automated insulin delivery (AID) systems; however, many people in the diabetes community have perceived the introduction of commercial AID systems to be overly slow (embodied in the #WeAreNotWaiting movement on social media).^1^ While commercial AID systems deliver reduced burden and improved glycemia,^2-4^ some regard these as not sufficiently customizable to meet their individual needs. This is reflected by the continued use open-source automated insulin delivery platforms globally, despite the increased accessibility of commercial systems.^1^

Despite large data sets from users of open-source AID systems showing glycemic efficacy^5^ and user satisfaction,^6^ many diabetes healthcare providers describe hesitancy regarding their use. One survey indicated that only 9% of such providers would initiate a conversation with their patients regarding open-source AID, but 47% would consider its use if they themselves had T1D.^7^

One major determinant for hesitancy in discussing open-source automation is the lack of regulatory approval and having been developed outside traditional models of evidence-based medicine. Rather than proceeding through conventional phase 1, 2, and 3 clinical trials, open-source AID was developed by individuals with T1D sharing their data, freely testing algorithm adjustments and making collective refinements to the code on this basis.^1^ Although fostering rapid innovation, this approach creates uncertainty regarding clinical risk and falls outside of any regulatory framework. It is understandable that clinicians may feel hesitant to recommend open-source AID when they feel unable to quantify the associated risk, and where there remain questions as to who should assume responsibility for that risk. Nevertheless, regulatory approval is possible for open-source system, as shown by Food and Drug Administration (FDA) approval for Tidepool Loop.^8^

The trial by Nanayakkara and colleagues^9^ assessed an open-source AID system AndroidAPS. In a crossover design, 20 adult participants used each of AndroidAPS and conventional insulin pump therapy (without CGM) for 4 weeks. The primary outcome, time in range (70-180 mg/dL) was significantly improved using AndroidAPS: mean 76.6% ± 11.7%, compared with 58.0% ± 15.6% with conventional pump therapy. These figures are similar to those reported in the 2022 CREATE trial, which also assessed AndroidAPS.^10^ Although there are no direct comparison data, it is notable that glycemic outcomes for AndroidAPS appear similar to pivotal trials of established commercial AID systems, including Control-IQ,^3^ Omnipod 5,^2^ and CamAPS.^4^

Importantly, Nanayakkara and colleagues did not observe any serious adverse events with use of AndroidAPS, including no episodes of severe hypoglycemia or diabetic ketoacidosis. Caution needs to be applied extrapolating to the real-world setting, given that this trial was of a relatively small number of participants using AndroidAPS for a short duration, while under close surveillance by a clinical team. However, longer studies of AndroidAPS have also shown safety.^10^ The growing body of evidence that AndroidAPS achieves similar glycemic outcomes to commercial AID systems provides reassurance that the relative risks and benefits are probably comparable, at least on average.

For the diabetes clinician, the validation of open-source AID in robust clinical trials provides two important messages. First, AndroidAPS is a safe and effective evidence-based therapy and its use should be supported in motivated people with diabetes who are willing to personally accept the risk associated with its lack of regulatory approval. Second, the growing data set from these trials support the notion that open-source AID can fall within usual clinical care.

While open-source AID will probably ever be used by only a minority of people with T1D, the efficacy of these systems is testament to the power of patient-driven innovation. Open-source AID is a leading example of the democratization of medicine.^11^ People with T1D should be central to all advances in diabetes care. Although the role of the clinician in maintaining patient safety cannot be overstated, an overly cautious approach to risk mitigation by the clinician may be interpreted by the patient-innovator as paternalistic. Management of T1D includes enablement of self-management by people with diabetes. Facilitating the skilled self-manager to build their own automated decision-making system is a logical continuation of this model.
